# Reconstruction Technique Options for Achieving Total Arterial Revascularization and Multiple Arterial Grafting

**DOI:** 10.3390/jcm12062275

**Published:** 2023-03-15

**Authors:** Dominique Vervoort, Malak Elbatarny, Rodolfo Rocha, Stephen E. Fremes

**Affiliations:** 1 Institute of Health Policy, Management and Evaluation, University of Toronto, Toronto, ON M5S 1A1, Canada; 2 Division of Cardiac Surgery, University of Toronto, Toronto, ON M5S 1A1, Canada; 3 Schulich Heart Centre, Sunnybrook Health Sciences Centre, Toronto, ON M4N 3M5, Canada

**Keywords:** cardiac surgery, coronary revascularization, coronary artery bypass grafting, arterial grafting

## Abstract

Ischemic heart disease is the leading cause of morbidity and mortality worldwide and may require coronary revascularization when more severe or symptomatic. Coronary artery bypass grafting (CABG) is the most common cardiac surgical procedure and can be performed with different bypass conduits and anastomotic techniques. Saphenous vein grafts (SVGs) are the most frequently used conduits for CABG, in addition to the left internal thoracic artery. Outcomes with a single internal thoracic artery and SVGs are favorable, and the long-term patency of SVGs may be improved through novel harvesting techniques, preservation methods, and optimal medical therapy. However, increasing evidence points towards the superiority of arterial grafts, especially in the form of multiple arterial grafting (MAG). Nevertheless, the uptake of MAG remains limited and variable, both as a result of technical complexity and a scarcity of conclusive randomized controlled trial evidence. Here, we present an overview of CABG techniques, harvesting methods, and anastomosis types to achieve total arterial revascularization and adopt MAG. We further narratively summarize the available evidence for MAG versus single arterial grafting to date and highlight remaining gaps and questions that require further study to elucidate the role of MAG in CABG.

## 1. Background

Ischemic heart disease remains the leading cause of morbidity and mortality worldwide. In 2019, 197 million people (95% uncertainty interval, UI: 178–200 million) lived with ischemic heart disease, 9.14 million people (95%UI: 8.40–9.74 million) died from ischemic heart disease, and 182 million disability-adjusted life years (95%UI: 170–194 million) were lost due to ischemic heart disease [1]. Coronary artery bypass grafting (CABG) is the preferred method of coronary revascularization for symptomatic multi-vessel coronary artery disease, particularly in patients with diabetes, complex coronary anatomy, or left ventricular dysfunction [2]. Although CABG volumes initially decreased over time due to the advent and development of percutaneous coronary interventions, its volume has stabilized in most high-income countries, and it remains the most common cardiac surgical procedure globally [3]. Over time, CABG has evolved with the exploration of different harvesting, anastomosis, and grafting choices and techniques, as well as lesser-invasive and off-pump approaches.

Saphenous vein grafts (SVGs) are the most frequently used bypass conduits for CABG, in addition to the left internal thoracic artery (LITA), is typically anastomosed to the left anterior descending artery (LAD). In the United States, an analysis of the Society of Thoracic Surgeons Adult Cardiac Surgical Database shows that approximately 80% of CABGs are performed with an anastomosis of the LITA to the LAD and anastomoses of SVGs for other coronary targets [4]. Long-term SVG patency has improved over time, especially due to secondary atherosclerotic prevention medications such as statins. However, observational data have shown greater patency and more favorable clinical outcomes if a second arterial graft is used as a bypass graft instead of an SVG [5]. Nonetheless, one of the limitations of multiple arterial grafting (MAG) is its greater technical complexity, with long-term mortality and the rates of deep sternal wound infections being directly influenced by surgeons’ expertise (i.e., greater proportional MAG use is associated with improved outcomes) [6,7,8,9]. This may be one reason why MAG volumes differ greatly between centers: for example, in the United States, adoption of MAG is lower in the South (~10% of CABG cases) compared to the Northeast (~20% of cases) [9].

In the current era, life expectancy is increasing, resulting in a larger patient population living with coronary artery disease. In addition, patients increasingly prefer less-invasive revascularization techniques. Surgical strategies such as left or bilateral thoracotomies and robotic CABG have emerged, particularly while using an off-pump technique with MAG or total arterial revascularization (TAR) with bilateral internal thoracic arteries (BITA). Nevertheless, the outcomes of minimally invasive CABG are not yet comparable to CABG via median sternotomy, especially with regard to the completeness of revascularization. Hence, continuous development of minimally invasive techniques is needed to reduce the need for reinterventions and improve long-term outcomes.

In this article, we present an overview of CABG techniques, harvesting methods, and anastomosis types to achieve TAR and adopt MAG. We narratively summarize the available evidence to date and highlight remaining gaps and questions that require further research.

## 2. Multiple and Single Arterial Grafting and Harvesting Methods

Arterial grafting has been extensively studied to date to determine short- and long-term outcomes stratified by the extent of arterial grafting (i.e., single- (SAG), multiple-, and total-arterial grafting (TAG)).

Observational evidence generally favors MAG over SAG. In Ontario, propensity-score matching of real-world data suggests that, at eight years, the risks of major adverse cardiac and cerebrovascular events (MACCE; Hazard Ratio, HR 0.82, 95% confidence interval [0.77–0.88]) and death (HR 0.80 [0.73–0.88]) were lower for MAG compared to SAG [10]. However, no differences in outcomes were found between patients managed with two (MAG) vs. three (TAG) arterial grafts. In women, propensity-score matched evidence suggests no differences in 30-day outcomes between MAG and SAG, whereas, at a median follow-up of five years, there was greater survival with MAG versus SAG (HR 0.85 [0.75–0.98]) and freedom from MACCE (HR 0.85 [0.76–0.95]) [11]. In the United States, however, low-MAG-volume centers show poorer outcomes compared to high-MAG-volume centers, suggesting important volume-outcome relationships [9].

Trial evidence evaluating arterial grafting has mounted over time. In the late twentieth century, Myers et al. [12] compared ITA-only MAG with grafting with ITA and SVG, finding comparable early and 5-year outcomes between both groups. Later, the Stand-in-Y Mammary Trial [13] found that MAG was superior to ITA-SVG. At two years, cerebrovascular and all-cause survival outcomes were comparable between MAG and SAG; however, cardiac event-free survival was lower after MAG compared to SAG, regardless of the arterial conduits or anastomotic techniques utilized. However, the Arterial Revascularization Trial (ART) [14,15,16] showed that BITA and SITA were associated with comparable outcomes at 10 years for both mortality (HR 0.96 [0.82–1.12]) and the composite of death, myocardial infarction, or stroke (HR 0.90 [0.79–1.03]).

Conflicting conclusions have been found in systematic reviews and meta-analyses, in part due to the discrepancy between the benefits found in observational studies and the conclusions drawn from existing trials. Gaudino et al. suggest that unmeasured confounding may be an important driver of the outcomes observed in cohort studies [17]. The Radial Artery International Alliance (RADIAL) study [18,19] pooled patient-level data from six randomized controlled trials (N = 1036; 534 radial arteries, 502 SVGs) to compare the use of the radial artery or the SVG in CABG. At a median follow-up of 10 years, the radial artery was associated with a reduced risk of the composite of death, myocardial infarction, or repeat revascularization (HR 0.73 [0.61–0.88]). Saraiva et al. [20] compared MAG and SAG by pooling data from 29 propensity-score-matched cohort studies and eight randomized controlled trials (N = 122,832; 52,178 MAG, 70,654 SAG). They found lower early mortality (OR 0.82 [0.71–0.95]), long-term mortality (HR 0.76 [0.73–0.78]), and MACCE (HR 0.85 [0.79–0.91]) for MAG compared to SAG [20]. MAG resulted in increased sternal wound complications when a BITA configuration was used (odds ratio: 1.96 [1.37–2.81]). Similarly, Urso et al. found that, while BITA was associated with a long-term survival benefit compared to SITA alone (HR 0.78 [0.71–0.86]), the addition of the radial artery to SITA resulted in comparable outcomes (HR 0.86 [0.69–1.07]) [21]. However, the risk of deep sternal wound infection after BITA was higher than after SITA (risk ratio 1.66 [1.41–1.95]). Further, Benedetto et al. evaluated the angiographic patency of the RITA, the radial artery, and the SVG through a network meta-analysis of nine randomized controlled trials (N = 145 RITA, 871 radial arteries, 845 SVGs): at follow-up, patency rates were superior for RITA compared to the radial artery, whereas both grafts were found to be superior to the SVG [22]. A more recent network meta-analysis by Deng et al. [23] of 18 trials with a total of 8272 grafts and a weighted mean angiographic follow-up of 3.5 years found that the radial artery (IRR 0.56 [0.43–0.74]) and no-touch SVG (IRR 0.56 [0.44–0.70]) were associated with reduced graft occlusion compared to the conventionally harvested SVG.

Based on the available evidence, current guidelines appear to favor MAG. The 2018 European Association for Cardio-Thoracic Surgery (EACTS)/European Society of Cardiology (ESC) guidelines [24] recommend MAG as Class IIa recommendations (Level of Evidence B). The more recent 2021 American Heart Association (AHA)/American College of Cardiology (ACC)/Society for Cardiovascular Angiography and Interventions (SCAI) guidelines [24] do not specifically comment on MAG but recommend radial artery use as Class I (Level of Evidence B-R), ITA use as Class I (Level of Evidence B-NR), and bilateral ITA (BITA) use as Class IIa recommendation (Level of Evidence B-NR). The 2016 Society of Thoracic Surgeons Practice Guidelines [4] are consistent with recommending MAG (Class IIa, Level of Evidence B) and provide more detailed recommendations on the choice and use of arterial conduits during CABG.

### Types of Arterial Grafts and Harvesting Methods

**Figure 1:**
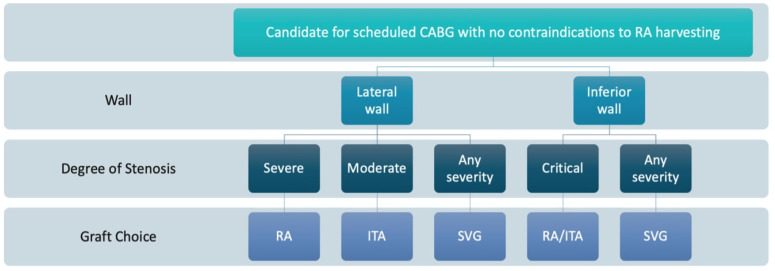
Flowchart for patient selection for single versus multiple arterial grafting in coronary artery bypass grafting (CABG). Adapted from Gaudino et al. [29]. ITA—internal thoracic artery; RA—radial artery; SVG—saphenous vein graft.

Several arterial grafts have been used to date (Figure 1). In the early 2000s, the ITA was used in approximately 92.4% of CABGs (4.0% BITA), with increasing use over time [25]. Today, rates are likely even higher, as supported by contemporary guidelines [26]. The graft is favored because of its optimal early- and long-term survival and patency rates. In addition, meta-analytic findings suggest that BITA use is associated with improved 10-year survival (HR 0.79 [0.75–0.84]) compared to single ITA use [26]. However, the use of both internal thoracic arteries is also associated with greater risks of deep sternal wound infection and mediastinitis, especially in patients who have obesity, poorly controlled diabetes, or chronic obstructive pulmonary disease [27,28]. As a result, BITA use is relatively contraindicated in patients with insulin-dependent diabetes mellitus, obesity, pulmonary disease, immunosuppression, steroid therapy, or prior chest irradiation [29]. In other patients, the low risk (<1%) of deep sternal wound infection and mediastinitis [30] is outweighed by the favorable long-term outcomes. Except in minimally invasive CABG, the ITA is harvested in an open manner, either pedicled or skeletonized [31].

The radial artery is the second-most commonly used arterial graft after the ITA because of the technical ease of harvesting the artery and its relatively large diameter compared to other arterial grafts. The artery is subject to anatomical variations in up to one in eight individuals [32,33], which may present as higher-than-normal origin, arterial tortuosity, and the presence of accessory branches. Contraindications are few but include complex anatomy, an abnormal Allen test, trauma (e.g., radial artery injury or dissection after cannulation, including radial access angiography), or ipsilateral breast surgery with axillary dissection, radiotherapy, and chronic lymphedema [34,35]. Additionally, in the case of end-stage renal failure, preservation of arteriovenous blood access for hemodialysis through the radial artery is necessary. In patients on hemodialysis, CABG is still associated with a reduced 5-year mortality compared to percutaneous coronary intervention [36], requiring careful graft selection. The estimated glomerular filtration rate is also an independent predictor of mortality after CABG [37]; as such, the radial artery may be spared in patients with or at high risk of renal failure. The radial artery has traditionally been harvested openly but is increasingly harvested endoscopically [38]. Endoscopic harvesting, when compared to traditional, open harvesting, reduces the incision size and is thus more cosmetically appealing and associated with reduced pain [39]. A recent meta-analysis of 18 trials (N = 2919; 1732 open harvesting, 1187 endoscopic harvesting) found that endoscopic harvesting was associated with lower rates of wound infection (RR 0.29 [0.14–0.60]) and neurological complications (RR 0.41 [0.27–0.62]) at the harvesting site, whereas the 30-day and 1-year mortality and graft patency rates were comparable [40].

The right gastroepiploic artery has very few contraindications [29], but its use is mostly limited to Japan and South Korea and is commonly used for anastomosis to the right coronary artery [41]. Centers from these countries have reported acceptable patency rates, particularly if harvesting is performed in a skeletonized fashion. For example, Suzuki et al. reported early and long-term outcomes and patency of patients undergoing off-pump CABG with an in situ skeletonized gastroepiploic artery as a graft [42]. Among 424 patients, 36.6% (N = 155, 215 anastomoses) received long-term follow-up with CT angiography. At a mean follow-up of 73 months, patency was 94.4%, ranging from 97.8% at 30 days and 96.7% at 1 year to 90.2% at 8 years. In particular, target vessel minimal lumen diameter on quantitative coronary angiography may be used to guide the choice of graft: Akita et al. report lumen diameters <1 mm as a predictor of long-term patency of the gastroepiploic artery (10-year patency rate of 89.8%) [43]. The use of the gastroepiploic artery as a graft has not been widely adopted in the rest of the world, although some reports suggest favorable outcomes. Angiographic predictors have been confirmed by prospective randomized comparisons in Belgium in the short- and mid-term, which further suggest pre-operative angiographic parameters may not influence SVG flow patterns to the same extent [44,45]. At three years, the SVG was associated with improved graft function compared to the gastroepiploic artery (OR 6.1 [2.4–15]) [45]. These findings may explain the lower use of the gastroepiploic artery globally; however, it is unclear whether the lower adoption thereof is responsible for the associated reduced patency (i.e., volume-outcome relationship) versus the result of anatomical or physiological differences. Indeed, propensity-score matching findings suggest improved long-term survival with the right gastroepiploic artery compared to the SVG as a third conduit in MAG with BITA: at 10 and 20 years, respectively, the survival was 98.9 ± 2 and 68.9 ± 18% for the gastroepiploic artery vs. 87.2 ± 4.6 and 50.3 ± 7% for the SVG as a third conduit [46].

Lastly, arterial grafts may be accompanied by SVGs, whose patency has improved over time as a result of novel techniques to harvest (e.g., no-touch) and preserve the graft [41]. An in-depth discussion of SVGs falls outside the scope of this article but has been covered in the recent literature [43].

The patency rates of different grafts have been evaluated in a multitude of studies, albeit often without direct head-to-head comparisons of all graft types. Gaudino et al. performed a network meta-analysis of randomized evidence comparing the angiographic patency rate (14 studies, 3651 arterial grafts, and SVGs) with a weighted mean angiographic follow-up of 5.1 years [47]. Graft occlusion was lowest for the radial artery (IRR 0.54 [0.35–0.82]) and no-touch SVG (IRR 0.55 [0.39–0.78]) when compared to traditionally harvested SVG, whereas it was higher for the RITA and gastroepiploic artery. Nevertheless, it must be acknowledged that the heterogeneity in study populations, center volumes, surgeon experience, study periods, and direct comparisons make it difficult to draw strong conclusions regarding long-term outcomes.

## 3. Techniques and Anastomoses for Arterial Grafting

MAG has been partially facilitated by incorporating composite grafts, which improve the efficiency of conduits, and various techniques of anastomosis. Composite grafting techniques include the use of T-shaped, Y-shaped, and I-shaped grafts [48,49,50]. Sequential grafting is also a key adjunct; anastomotic technical variations include side-to-side, end-to-side, terminolateral, laterolateral, and diamond-shaped anastomoses [50]. Beyond improving the efficiency of each additional conduit, facilitating anaortic CABG, and extending the flexibility to address complex lesion configurations, there are theoretical hemodynamic benefits to incorporating various configurations of conduits into an operation [50,51]. For example, the construction of sequential grafts using diamond-shaped configurations to optimize graft length is preferred over laterolateral anastomoses when coronary targets are close together due to the risk of kinking [50]. It is believed that diamond-shaped and side-to-side anastomotic configurations may be beneficial in small target arteries due to increased coronary flow compared to end-to-side configurations [51]. In a small case series comparing small terminal coronary targets revised from end-to-side to terminal side-to-side configuration for poor graft flow, there was a highly significant four-fold increase in intraoperative graft flow [52]. However, this series only examined patients with SVGs, and technical causes of initial poor flow were not definitively ruled out [52]. Additionally, clinical significance (i.e., impact on long-term patency) was outside of the scope of the study. Incorporating sequential side-to-side anastomoses may improve blood flow in side-to-side targets, whereby distal segments may be more sensitive to competitive flow and require a lower fractional flow reserve for functional patency [53]. In other intraoperative analyses of blood flow comparing coronary graft flow with side-to-side patent sequential anostomoses with flow under temporary occlusion, there were no differences in terminal coronary flow in left- [54] or right-sided targets [55], highlighting the need for further study. These hemodynamic data suggest that composite grafts may be comparable to non-composite grafts at intraoperative assessment and potentially superior in selected circumstances.

The use of ITA and radial conduits in sequential configurations involves greater technical complexity compared to SVGs; nevertheless, excellent clinical results have been demonstrated in the literature [56,57,58,59]. The patency of sequential LITA to LAD and diagonal is over 98% and non-inferior to separate LITA grafting, with greater than 99% patency of the first target and greater than 97% patency of the second target at 2.8 years [58,59]. Furthermore, symptom-driven angiographic studies show similar risks of ITA graft occlusion in two large series of primarily in situ and primarily Y-configuration ITA-CABG [60,61,62]. In a systematic review of four randomized trials and three observational studies [63], no differences in long-term survival or major adverse cardiovascular events (MACE) were identified between in situ versus the Y configuration of BITA. Only one observational study showed a marginal difference in freedom from repeat revascularization between composite and non-composite configurations [in situ LITA-LAD RITA-Cx 93.9 vs. in situ LIMA-LAD RITA-RCA 90.7 vs. RITA-LAD LITA-LCx 85.1 vs. RITA-free Y-graft 87.5 Log Rank P = 0.049] [64]. Among 436 angiograms performed at a mean of 35 months after CABG involving I- or Y-sequential radial grafts, 96% were patent to all targets [48]. Similarly, in the Stand-in-Y study, the anastomotic configuration did not influence clinical outcomes [49]. A major consideration to minimize the risk of occlusion is the degree of stenosis of targets, thus avoiding the risk of competitive flow. The Impact of Preoperative Fractional Flow Reserve on Arterial Bypass Graft Function (IMPAG) trial was the first to identify that preoperative fractional flow reserve predicts graft function at six months [53]. There was no difference between sequential or Y-grafts compared to non-composite configurations, confirming that target stenosis is likely a higher priority consideration than graft configuration [53]. Similarly, in radial composite grafts, target vessel stenosis remains the most important predictor of late patency as opposed to anastomotic configuration [48]. Much like in situ ITA, ITA Y-grafts increase in diameter within the first year of grafting, and this occurs proportionally to the degree of target stenosis [65]. The adaptability of both in situ and Y-ITA implies that a single ITA can adapt to provide sufficient inflow for large territories of the myocardium. Hemodynamic studies show that both in situ and T/Y-grafted ITAs provide diastolic-predominant peak flow at the coronary side, mimicking normal physiology [66,67]. Interestingly, only T/Y-grafts seem to display this pattern close to the subclavian end as well [66,67]. The clinical significance of these flow differences, if any, remains unknown.

A Y-composite of the no-touch SVG and LITA has been explored with favorable results. Kim et al. [68] evaluated competitive flow results on post-operative angiograms early after surgery and, if present, one year after revascularization. Among 806 patients, 11.7% (N = 94) showed competitive flow after surgery (no-touch SVG competition in 74 patients; LITA competition in 20 patients). At one year, 63 patients (50 with primary SVG competition and 13 with LITA competition) were followed up with angiograms: 80% of the no-touch SVG conduits with the competitive flow were patent, whereby the rates of perfect patency of no-touch SVGs with pedicle tissue were greater compared to those of no-touch SVGs without pedicle tissue (42.5% vs. 12.5%, *p* = 0.007).

Lastly, anaortic or no-touch aortic surgery eliminates the need for cardiopulmonary bypass and reduces aortic manipulation. It has been suggested that such an approach may reduce the risk of post-operative stroke, which remains a concern with CABG. A network meta-analysis comparing anaortic off-pump CABG, clampless off-pump CABG, partial-clamp off-pump CABG, and traditional CABG with aortic cross-clamping evaluated outcomes in 37,720 patients across 13 studies [69]. Anaortic off-pump CABG was found most effective in reducing post-operative stroke risks (OR 0.22 [0.14–0.33] vs. CABG; OR 0.34 [0.22–0.52] vs. partial-clamp off-pump CABG; OR 0.48 [0.27–0.86] vs. clampless off-pump CABG) as well as early mortality, renal failure, bleeding complications, atrial fibrilation, and intensive care unit length of stay. However, the meta-analysis was unable to discern the completeness of revascularization and patency rates of the underlying studies, as well as account for different levels of surgeons’ technical experience with off-pump CABG techniques [70]. More recently, Albert et al. reported on a large (N = 4485 by 18 surgeons) single-center experience of using the anaortic off-pump CABG approach [71]. Compared to patients receiving on-pump CABG, the post-operative stroke rate was lower (0.49% vs. 1.31%, *p* < 0.0001) as a result of reduced early (but not delayed) strokes (0.09% vs. 0.83%, *p* < 0.0001) when undergoing the anaortic approach. Findings were consistent in a smaller sample analyzed with propensity-score matching. However, similarly, revascularization and long-term mortality were not evaluated, which have been of concern in off-pump CABG trials [72]. Thus, further study is needed to best evaluate anaortic and off-pump CABG techniques compared to traditional CABG.

Overall, intraoperative, hemodynamic, and clinical outcomes data demonstrate the safety of various composite arterial graft configurations and anastomotic techniques. These represent important tools to increase the adaptability of MAG and address a broad range of complex coronary anatomical substrates.

## 4. Remaining Gaps and Questions

Various gaps and questions remain. First, observational data have shown that MAG may be associated with improved outcomes in the general population when compared to SAG. However, the benefit of MAG in specific patient populations remains to be elucidated. In the few retrospective reports comparing MAG and SAG in women, MAG had significantly better outcomes compared to SAG [11,73,74]. In patients with diabetes, MAG was also associated with improved outcomes, although at the expense of a higher risk of deep sternal wound infection when BITA was used [75]. In patients with end-stage renal disease, the use of the radial artery is not advised because of its use for arteriovenous blood access, whereas the use of the SVG must be balanced against the risk of peripheral arterial disease in this patient population, often necessitating SVGs for lower limb bypass surgery [76]. In patients on hemodialysis, for example, the presence of clinically relevant peripheral arterial disease is as high as 15% [77]. Meanwhile, the use of BITA versus SITA in patients on hemodialysis was not associated with an increased risk of mediastinitis or a difference in early mortality [78]. Most studies have compared MAG to SAG in a younger population that may benefit from improved long-term outcomes (10 years or more post-index procedure) [79]. Yet, the influence and potential long-term benefit of MAG in patients older than 70 years have not been widely investigated [80]. Finally, there is limited evidence on the outcomes of MAG with the use of the radial artery in patients with moderate-to-severely reduced ventricular function, although it does appear safe when used selectively [81]. Further research may be particularly interesting to evaluate since this sub-group of patients may require higher doses of inotropes and vasopressors in the postoperative period, potentially increasing the risk of radial artery vasospasm and acute coronary events [35].

**Figure 2:**
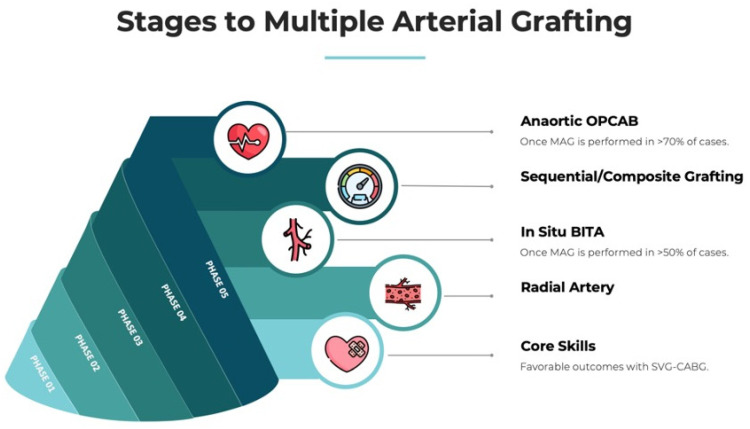
Integrating multiple arterial grafting (MAG) in coronary artery bypass grafting (CABG) programs. Adapted from Vallely et al. [88]. BITA—bilateral internal thoracic artery; OPCAB—off-pump CABG; SVG—saphenous vein graft.

Second, one of the Achilles heels of MAG remains the technical expertise required to reproduce the procedure with adequate short- and long-term success. There is an ongoing debate about whether surgical coronary revascularization should be a cardiac surgery subspecialty such as aortic or mitral surgery [82,83]. This is particularly topical considering the rapid growth and adoption of more novel CABG techniques, including off-pump, anaortic, minimally invasive, and robotic CABG. Indeed, institutions are increasingly searching for coronary revascularization experts to safely perform off-pump cases for patients with a porcelain aorta or MAG/TAR for young patients with very long life expectancies (Figure 2).

Third, most reports of MAG and TAR are still observational and, therefore, have inherent selection bias and unmeasured confounding [77,78]. The Randomization of Single vs. Multiple Arterial Grafts Trial (ROMA; NCT03217006) [84,85] will compare different revascularization modalities with diligent criteria for patients (i.e., <70 years of age) and surgeons (i.e., low conversion from one revascularization modality to another) to minimize the effect of confounders. Furthermore, the ROMA trial will be extended to investigate the revascularization outcomes of women (Randomized Comparison of the Outcomes of Single vs. Multiple Arterial Grafts in Women (ROMA:Women; NCT04124120)). Finally, global initiatives facilitating multi-institutional reports of expert centers, such as the Arterial Grafting International Consortium (ATLANTIC) Alliance, may allow for more significant insights regarding the optimal mode of surgical revascularization for each specific patient [86,87].

Lastly, the role of complex coronary surgery, whether through an off-pump technique [88], a minimally invasive direct coronary artery bypass, or robotic approaches, remains to be determined. In particular, there remain concerns regarding the completeness of revascularization when using less-invasive modalities, which require further study and technical improvements. In addition, the advantages of less widely adopted arterial grafts, such as the right gastroepiploic artery, are still uncertain beyond settings and centers where their adoption has been widespread.

## 5. Conclusions

The majority of evidence to date supports the roles of TAR and MAG but remains largely based on observational data, which are limited by a high risk of confounding. Similarly, past clinical trials were underpowered to study the comparative effectiveness of TAR, MAG, and SAG and showed conflicting findings. The upcoming ROMA and ROMA:Women trials, which are powered for this particular question, will shed further insights into the roles of MAG and TAR. Nevertheless, while arterial grafting appears promising, continued scrutiny is necessary to (1) ensure sustained improvements in the long-term patency of SVGs, especially considering their wide adoption in CABG procedures around the world, (2) optimize patient selection for different treatment options in terms of sex, age, comorbidities, and other relevant determinants, and (3) consider the role of surgeons’ and centers’ experience when considering more complex revascularization strategies in light of volume-outcome relationships.

## Data Availability

Not applicable.
